# Acknowledgement to Reviewers of *Children* in 2019

**DOI:** 10.3390/children7010007

**Published:** 2020-01-16

**Authors:** Children Editorial Office

**Affiliations:** MDPI, St. Alban-Anlage 66, 4052 Basel, Switzerland

The editorial team greatly appreciates the reviewers who have dedicated their considerable time and expertise to the journal’s rigorous editorial process over the past 12 months, regardless of whether the papers are finally published or not. In 2019, a total of 136 papers were published in the journal, with a median time to first decision of 26 days and a median time from submission to publication of 46 days. The editors would like to express their sincere gratitude to the following reviewers for their generous contribution in 2019:
Abbott, DavidLi, LiAbsoud, MichaelLin, WenAddo, O. YawLindqvist, Anna-KarinAfolayan, Adeleye J.Logan, DeirdreAgoston, MonicaLovvorn, HaroldAgrawal, HiteshMacLeod, StuartAkram, GazalaMagriplis, EmmanuelaAllegaert, KarelMair, JacquelineAllen, LoydMarchak, Jordan GillelandArlati, SaraMarchesi, AlessandraAsbrand, JuliaMareci, Thomas H.Aslanidis, TheodorosMarron, JonathanBaak, MelanieMartin, NicolaBackes, ToddMartínez-Ferrer, BelénBaker, Mark DouglasMathew, MonaBallweg, Jean A.Mdegela, MselengeBalwicki, ŁukaszMeany, HollyBarbi, EgidioMeghdari, AliBarbosa, Carlos MauricioMisra, SanghamitraBarnes, AndrewMiyagi, HisayukiBaroncini, DamianoMiyoshi, TakekazuBartkowski, JohnMohd, Farooq ShaikhBekx, TracyMorales, SantiagoBelteki, GusztavMorseth, BenteBender, Hans-UlrichMuensterer, Oliver Hans - JosefBergen, DorisMuñoz, Cynthia E.Bickel, ScottMupo, AnnalisaBidzan, MariolaNapolitano, MaddalenaBiglino, GiovanniNarayan, AngelaBinford, WarrenNavarro-Lopez, VicenteBoyce, Lisa K.Navidi, UteBoykan, RachelNehlig, Astrid Y.Bradley, David J.N’Gouemo, ProsperBrock, KatharineNguyen, ThinhBrzeziński, MichałNicklas, TheresaBuelga, SofíaNicolini, AntonelloBurton, Richard F.Niemi, Anna KaisaButcher, LucyNilsson, StefanCampbell, MarilynNir, AmiramCangiano, BiagioNoel, Gary J.Carotenuto, MarcoNoritz, GareyCasanovas, CarmenNorona, Amanda N.Cecchin, Frank C.O’Grady, LynCensani, Marisa A.Oberg, CharlesChalah, Moussa AntoineOkhuysen-Cawley, ReginaChandrasekharan, Praveen K.Olivares-Olivares, Pablo JoséChang, Mary P.Oliveira, GabrielleChen, WenchunOliver, JaneChui, KennethOllech, AyeletCianchetti, CarloOrtiz-Colón, Ana MaríaClementi, MichellePagano, IanCollongues, NicolasPapaioannou, Vasilios ECorradin, MarcoPapetti, LauraCree-Green, MelanieParrish, RichardCrock, MaryPease, Anna S.Crowe, SuzannePensabene, LiciaCzech, IwonaPerdikaris, PantelisDahdah, NagibPereira-da-Silva, LuisDanforth, Teresa L.Pergert, PernillaDelair, ShirleyPeterson, CatherineDering Anderson, AllyPeterson, GregoryDeUgarte, Daniel A.Pielech, MelissaDigidiki, VasileiaPietrzak, AldonaDionne, AudreyPistelli, FrancescoDoshi, Arpan R.Pizzorno, Mario AndresDouglas, FloraPolina, IuliiaDriessen, Gertjan J.Pratesi, SimoneDunphy, KimPrincipe, Maria Ilaria DelEl Manouni El Hassani, SofiaPsihogios, Alexandra M. PsihogiosEttekal, IdeanPulakka, A.Eyles, DarrylPulignani, SilviaFabbrocini, GabriellaPuri, KritiFenlason, Lindy ChristineQureshi, AtharFingerhut, RalphRawat, MunmunFinn, DaraghReid, KathyFish, Jonathan D.Remy, Kenneth E.Ford, JenniferRieder, MichaelFox, Claudia K.Riley, Carley L.Fredricks, Karla MarieRintala, Risto JFrew, John W.Roberts, Michael WFröberg, AndreasRoccella, MicheleGanapathy, VengateshRodeberg, DavidGarcia, Oscar FRodrigo, LuisGarcía, FernandoRodríguez-Almagro, Julián JavierGarcía, OscarRomero-Rodríguez, Luis MiguelGarcia Sanz, JoseRomo-Palafox, Maria JGaspar, NathalieRose, KlausGazmuri, Raúl J.Rossman, IanGebhardt, MarkusRugytė, DanguolėGerhold, KerstinRus, Horea G.Gershan, LynnRusso, EthanGianfrancesco, Milena A.Sadak, Karim ThomasGill, TiffanySaluk-Bijak, JoannaGinty, Annie T.Samadi, Sayyed AliGiuliani, CristinaSandhu, Satinder K.Giuliano, Ryan J.Schacter, Hannah L.Gomula, AleksandraSchapiro, NaomiGor, BeverlySchmoelzer, GeorgGorini, FrancescaSchultz, Kris Ann P.Gorodzinsky, AyalaSegal, UmaGreenough, AnneSeitz, ChristopherGroner, Judith A.Sekar, Krishnamurthy CGudena, Vinay KumarShaffner, Donald H.Guo, Chao-YuShah, BirjuGupta, NehalSiegel, Robert M.Gupta, AnshuŠileikienė, RimaHa, Min-SeongSilva, SusanaHanff, L.M.Sime, DanielaHaring, RobinSimeon, James C.Harrington, Kathleen F.Singer, DominiqueHeathcote, Lauren C.Skrzypiec, GraceHerbert, AnthonySnyder, Christopher S.Hernáiz Driever, PabloSotgiu, StefanoHeston, Thomas FSpiegler, JulianeHidalgo Lopezosa, PedroSt Hilaire, Melissa A.Hildreth, BlakeStachenfeld, NinaHill, Douglas L.Stein, JohnHughes, Jean R.Storcksdieck Genannt Bonsmann, StefanHutchon, DavidStrickley, RobertHwu, Wuh-LiangSuleman, ShazeenIlluminati, GiulioSulman, Cecille GIlyas, MohammedTambyraja, Sherine R.Imamura, MasahiroTanaka, NaoroIrtan, SabineTaverna, LiviaItkonen, TiinaTeicher, Carrie LeeJaaniste, TiinaTemple, LizJacobs, ShanaTeng, Ru-JengJaekel, JuliaTettenborn, BarbaraJain, SameerThomas Tobin, CourtneyJalava, KatriThompson, Deanne K.Jelin, EricTisdall, KayJeong, Se KyooTomasi, Paolo A.Jerger, KristinTremolada, MartaJeronen, EilaTrevisanuto, DanieleJeżewska-Zychowicz, MarzenaTryggestad, JeanieKabiri, Laura S.Vali, PayamKaiser, PamelaVallone, DonnaKatheria, AnupVan Tilburg, Miranda A.L.Kaushik, ShivaniVan Woerden, IreneKeller, BradleyVargas, Jorge H.Kim, John S.Velisek, LiborKirkegaard, Emil O.W.Versacci, PaoloKlapper, PaulVesoulis, Zachary A.Kleinert, EvelynVišnjić, AleksandarKobbe, RobinVisser, RenskeKoch, KatherineVoges, IngaKokkalera, Stuti S.Wallace, DustinKorcz, AgataWanchai, AusaneeKorpe, PoonumWang, XinwenKrajnović, DušankaWatson, Silvana Maria RussoKukem, Tesfaye SemelaWay, Denise AnnieKumar, VasanthWenz-Gross, MelodieLakshminrusimha, SatyanWhitaker, Amanda T.Lammert, CraigWhite, Rhiannon L.Lang, DiWilliams, Cydni N.Lau, Erica Y.Winestone, LenaLazaro Gutierrez, RaquelWiswell, Thomas E.Lee, Young TakWoolcock, GeoffreyLee, Chih-HungWright, SukhvirLee, A-WYamada, Nicole K.Lee, CarltonYanda, MuraliLee, Henry C.Zeng, NanLempart, LaurenZhao, WenbingLevy, RonaZwi, Karen

